# Startling Mosaicism of the Y-Chromosome and Tandem Duplication of the *SRY* and *DAZ* Genes in Patients with Turner Syndrome

**DOI:** 10.1371/journal.pone.0003796

**Published:** 2008-11-24

**Authors:** Sanjay Premi, Jyoti Srivastava, Ganesan Panneer, Sher Ali

**Affiliations:** Molecular Genetics Laboratory, National Institute of Immunology, Aruna Asaf Ali Marg, New Delhi, India; Institute of Genomics and Integrative Biology, India

## Abstract

Presence of the human Y-chromosome in females with Turner Syndrome (TS) enhances the risk of development of gonadoblastoma besides causing several other phenotypic abnormalities. In the present study, we have analyzed the Y chromosome in 15 clinically diagnosed Turner Syndrome (TS) patients and detected high level of mosaicisms ranging from 45,XO:46,XY = 100:0% in 4; 45,XO:46,XY:46XX = 4:94:2 in 8; and 45,XO:46,XY:46XX = 50:30:20 cells in 3 TS patients, unlike previous reports showing 5–8% cells with Y- material. Also, no ring, marker or di-centric Y was observed in any of the cases. Of the two TS patients having intact Y chromosome in >85% cells, one was exceptionally tall. Both the patients were positive for *SRY*, *DAZ*, *CDY1*, *DBY*, *UTY* and *AZFa*, *b* and *c* specific STSs. Real Time PCR and FISH demonstrated tandem duplication/multiplication of the *SRY* and *DAZ* genes. At sequence level, the *SRY* was normal in 8 TS patients while the remaining 7 showed either absence of this gene or known and novel mutations within and outside of the HMG box. SNV/SFV analysis showed normal four copies of the *DAZ* genes in these 8 patients. All the TS patients showed aplastic uterus with no ovaries and no symptom of gonadoblastoma. Present study demonstrates new types of polymorphisms indicating that no two TS patients have identical genotype-phenotype. Thus, a comprehensive analysis of more number of samples is warranted to uncover consensus on the loci affected, to be able to use them as potential diagnostic markers.

## Introduction

Turner Syndrome (TS), the common genetic abnormalities affecting ∼1 in 1500–2000 live female births [1]–[3], is suggested to be due to absence of the second X chromosome in part or full [3]–[4]. However, in ∼3–6% cells, the second sex chromosome is Y [5]–[6] that often triggers development of gonadoblastoma [7]. The Y chromosome in Turner patients is structurally abnormal showing deletions, inversions, dicentrics and ring forms [8]–[9] and becomes unstable resulting in 45/XO karyotype. Chromosomal constitution influences phenotypic sex and 45, XO cell line is frequently detected in males with gonadal dysgenesis in addition to TS patients [10]–[11]. It is largely believed that no two TS patients are identical with respect to the number of Y bearing cells or Y-linked loci. Moreover, this mosaicism varies across the tissues and thus accurate interpretation depends upon the number of cells analyzed and tissues selected [12]–[13]. The phenotypic sex is under the influence of Y chromosome and expression of Y linked loci in gonads [14]–[15]. In several instances, gonadectomy is conducted due to increased risks of gonadoblastoma [16]. However, actual distribution of the Y chromosome in tissues of the TS patients and its role remain a murky proposition.

Present study was conducted to investigate molecular alterations in the Y-linked loci in 15 clinically diagnosed TS patients. We detected large scale Y chromosome mosaicism ranging from pure 45/XO conceptus to ∼90% cells positive for an intact Y chromosome and XXX, XYY, XXY constitutions. Further, several Turners patients showed tandemly arranged multiple copies of the *SRY* and *DAZ* genes in addition to known and novel *SRY* mutations within and 5′/3′ regions of the HMG box.

## Results

### Turner Karyotypes and the Y chromosome

**Figure 1:**
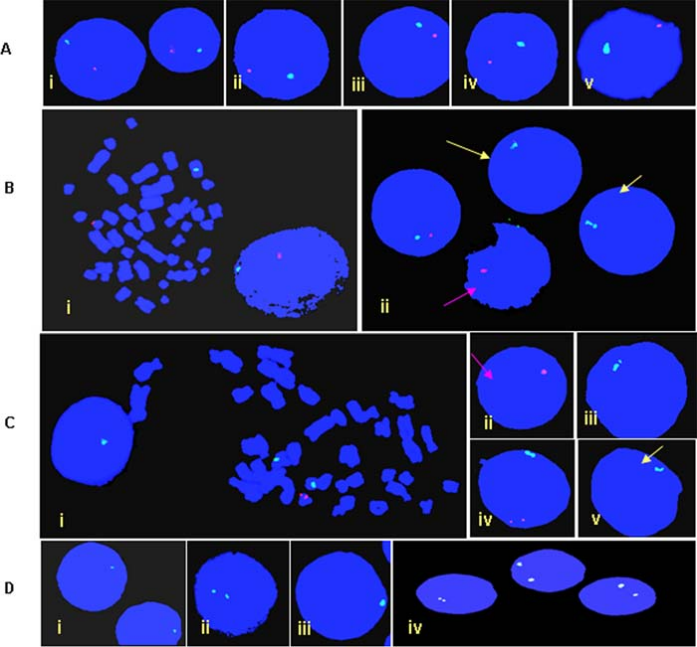
Fluorescence *in-situ* hybridization (FISH) using LSI *SRY* probe from VYSIS (which binds simultaneously to the *SRY* gene and centromere of the X chromosome) within the interphase nuclei and metaphase chromosomes of Turner AT1. (A i–iv) shows presence of both X (green dot) and Y (red dot) chromosomes in the interphase nuclei. Note structurally normal Y chromosome and absence of ring or dicentric one in (Bi) and (Ci) where the *SRY* gene is localized on the Yp. Some cells showed absence of the X chromosome, denoted by pink arrows (Bii) and (Cii). The classical Turner karyotypes (45, XO) are shown by yellow arrows. Some cells showed 47, XYY (Civ). (D), Cells without Y but variable numbers of X chromosome ranging from 1 (45, XO) to 2 (46, XX) were also detected. Only representative cells with different karyotypes are shown here. Single localized signal of the *SRY* gene (copy number 16) in AT1 suggests tandem duplication of this gene.

**Figure 2:**
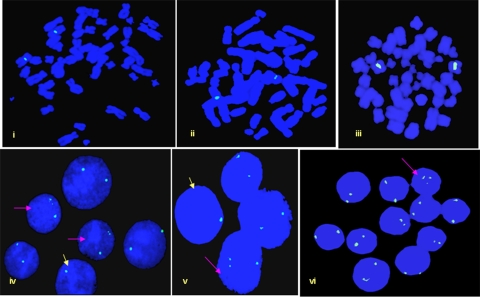
FISH with interphase nuclei and metaphase chromosomes of a Turner patient (AT4) with LSI-*SRY* probe. Note absence of the *SRY* signals in all the cells. No detectable Y chromosome at the level of PCR or G-banding was detected in this patient. The alterations detected in the number of X-Chromosomes are indicated by arrows. Pink arrows show cells with three X-Chromosomes (47, XXX) and the yellow ones highlights the cells with a single X-Chromosome (45, XO). Remaining interphases showed two X-Chromosomes (46, XX). Analysis of metaphase chromosomes (i–ii) further substantiated absence of the Y-chromosome. This is in contrast to Turner AT1 where >80% cells harbored Y-chromosome.

TS patients analyzed were in the age group of 14–25 yrs. Presence of Y chromosome detected with G-Banding was confirmed by FISH with Y specific probes *SRY*, 46A6 (*DAZ*) and 336F2 (gr/gr AZFc amplicons). Patients showed two extreme karyotypes, ones with >85% cells harboring Y chromosome (AT1 and AT15) and others with almost negligible presence of the Y chromosome (AT4, AT5, AT6 and AT7) (Table 1). Marker, ring or dicentrics Y chromosome was not observed in any of the patients. Clinically, all the TS patients had webbed neck, shield like chest and other characteristic features (Table 1) but no symptoms/trace of gonadoblastoma. Only one Turner (AT9) showed dysgenic testis which was removed surgically. One TS patient (AT15) was exceptionally tall with a normal female phenotype, but with clinical features similar to that of the Turners'. In addition to normal mosaic karyotypes, two Turners', AT1 and AT15 also showed another cell lines with 47, XYY, or 47, XXY chromosomal constitutions (Figure 1). Pure XO conceptus was also detected in some Turners, showing two, one or no signal for X chromosome but none at all for the Y chromosome (Figure 2).

**Table 1:** Details of the karyotypes and hormonal profiles of different Turner patients analyzed^#^

Patients	Phenotype	Karyotype (% cells)	Clinical Features	Hormonal Profiles
**AT1**	F	46,XX (10):46,XY (75):45,XO (5):47,XXY (2):47,XYY(3)	Age = 14 years, menarche not attained yet, secondary sex characters not developed, external genitalia normal, puberty grade 0, aplastic uterus, ovaries not seen, shield like chest, no skeletal anomaly, webbed neck	LH = 16.2 U/L, FSH = 89.8 U/L, PRL = 10.4 µg/L, TSH = 1.7 m U/L
**AT2**	F	46,XX (77): 46,XY (6):45,XO (14): 47,XXX (3)	Primary amenorrhea, atopic vagina, small uterus seen during gynecological examination, no endometrial tissue seen in biopsy, USG showed anteverted and ante flexed uterus	LH = 22 U/L, FSH = 75–80 U/L, PRL = 12 µg/L, TSH = 1.2 m U/L
**AT3**	F	46,XX (2):46,XY (3):45,XO (95)	Primary amenorrhea, small nodule like uterus but no ovaries, small stature and webbed neck	LH = 18 U/L, FSH = 75 U/L, PRL = 9 µg/L, TSH = 1.2 m U/L
**AT4**	F	46,XX (0):46,XY (0):45,XO (100)	Short stature, webbed neck, hypoplastic uterus and ovaries, normal external genitalia, underdeveloped breasts	LH = 32.0 U/L, FSH = 3.0, PRL = 14.0 µg/L
**AT5**	F	46,XX (5):46,XY (0):45,XO (95)	Webbed neck, nodule like uterus, Primary amenorrhea, under developed sexual characters	LH = 20 U/L, FSH = 45 U/L, PRL = 18 µg/L, TSH = 1–1.5 m U/L
**AT6**	F	46,XX (0):46,XY (0):45,XO (100)	Primary amenorrhea, small under developed uterus, no ovaries seen	NA
**AT7**	F	46,XX (5):46,XY (0):45,XO (95)	NA	NA
**AT8**	F	46,XX (5):46,XY (3):45,XO (92)	Turner variant, Primary amenorrhea, extremely hypoplastic uterus, breast and external genitalia were underdeveloped	LH = 37 U/L, FSH = 89.8 U/L, PRL = 10.4 µg/L, TSH = 1.7 m U/L
**AT9**	F	46,XX (5):46,XY (5):45,XO (90)	Short stature, webbed neck, primary amenorrhea, dysgenic gonads, testis were surgically removed	FSH = 35 U/L, PRL = 10.4 µg/L, TSH = 1.7 m U/L
**AT10**	F	46,XX (5):46,XY (3):45,XO (92)	Primary Amenorrhea	FSH = 90 U/L, PRL = 18 µg/L
**AT11**	F	46, XX (2):46, XY (2):45, XO (96)	Primary amenorrhea, webbed neck, no skeletal deformation, external genitalia normal, underdeveloped breasts	NA
**AT12**	F	46, XX (5):46, XY (3):45, XO (92)	Webbed neck, short stature, poorly developed secondary sexual characters	LH = 55–80 U/L, FSH = 85 U/L, PRL = 10–15 µg/L, TSH = 2 m U/L
**AT13**	F	46, XX (40):46, XY (30):45, XO (20)	Short stature, webbed neck, shield like chest, secondary amenorrhea, under developed breasts, external genitalia normal, USG showed streak gonads.	NA
**AT14**	F	46,XX (5):46,XY (3):45,XO (92)	Short stature, webbed neck, shield like chest, no breast nodules	LH = 56 U/L, FSH = 85 U/L, PRL = 20 µg/L, TSH = 1.5–2 m U/L
**AT15**	F	46,XX (5):46,XY (80):45,XO (5):47,XXY (2):47,XYY(3)	Bone age 25 years, height = 175 cm, weight = 50 kg, no skeletal deformation, Secondary amenorrhea, external genitalia normal, aplastic uterus, no ovaries observed except streak gonads.	LH = 25 U/L, FSH = 92 U/L, PRL = 8 µg/L, TSH = 2.0 m U/L
**NM**	M	46, XY	Normal	LH = 0.007–0.024 U/L, FSH = 5–20 U/L
**NF**	F	46XX	Normal	LH = 5–20 U/L, FSH = 3–20 U/L, PRL = 10–25 µg/L

# NM = Normal male, NF = Normal female, NA = Not available, USG = Ultrasonography, PRL = Prolactin releasing hormone, TSH = Thyroid stimulating hormone, FSH = Follicle stimulating hormone, LH = Luteinizing hormone. Numbers in parenthesis under karyotype denote percentage of the cells with the particular chromosomal constitution. All the karyotypes were confirmed analyzing ∼400 metaphase chromosome sets per individual.

### Structural integrity of Y the chromosome

**Figure 3:**
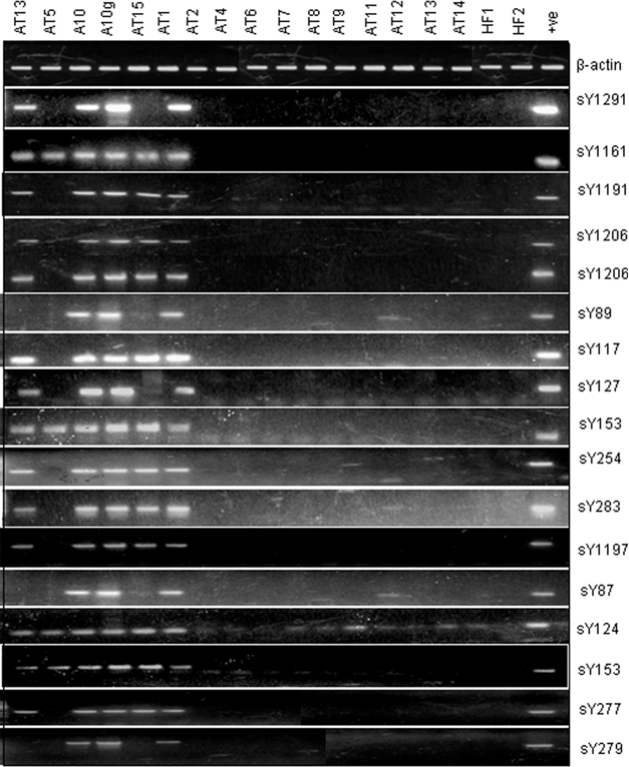
Representative gels showing STS mapping of the Y chromosomes in TS patients. STSs used are given on the right and sample IDs on top. The IDs ‘AT’ are Turners and their details are given in the table 1. A10 and A10g represent blood and semen DNA samples, respectively, from a single azoospermic male. HF denotes human female DNA sample. β-actin primers were used to normalize the quality and quantity of DNA used as template in PCR. Note presence of most of the STSs in Turners AT1 and AT15. Some STSs were positive in case of Turner AT13 as well but owing to non-availability of the fresh blood, the FISH experiments could not be conducted (see Tables 1 and 2 for details of the Turner patients).

**Figure 4:**
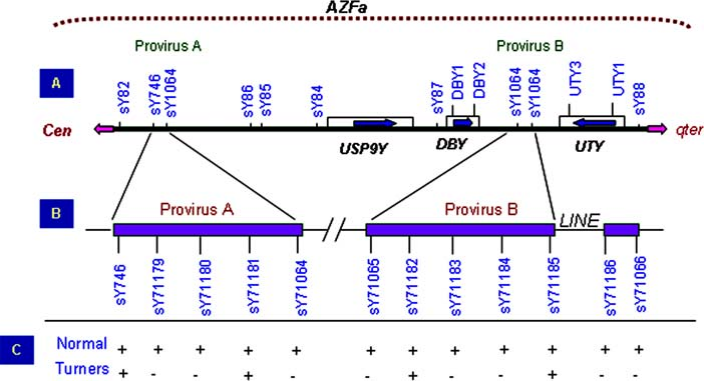
Analysis of the *AZFa* region of the Y chromosome in Turner Patients for possible HERV mediated recombination. (A) *AZFa* region of the human Y chromosome indicated as horizontal bar with centromere towards left and Yq to right. Various STS markers used for the analysis of the *AZF*a region and the candidate genes (*DBY*, *UTY*, *USP9Y*) are mentioned in the figure. The positions of provirus element A and B are shown by red dotted lines. (B) Detailed structure of the provirus A and B. Note the LINE insertion in provirus B. Various STS markers used to assess recombination events involving provirus elements are also indicated. (C) Results of the provirus (HERV) mapping of the Turner's syndrome. It may be noted that none of the males showed characteristic patterns of HERV mediated recombination leading to the AZFa deletion or duplication.

Structural integrity of the Y chromosome was assessed by routine STS mapping. In TS patients with more than 40% cells positive for Y chromosome, most of the STS's used were positive (Figure 3). STS mapping nullified any event of the gr/gr or b1/b3 major deletion phenotypes [17]. STSs lying in the crucial regions like *DAZ* gene, HERV sequences, AZF boundaries and other crucial genes were found to be intact except few randomly scattered microdeletions (Figure 4).

### Tandem duplication of the *SRY* and *DAZ* genes

**Figure 5:**
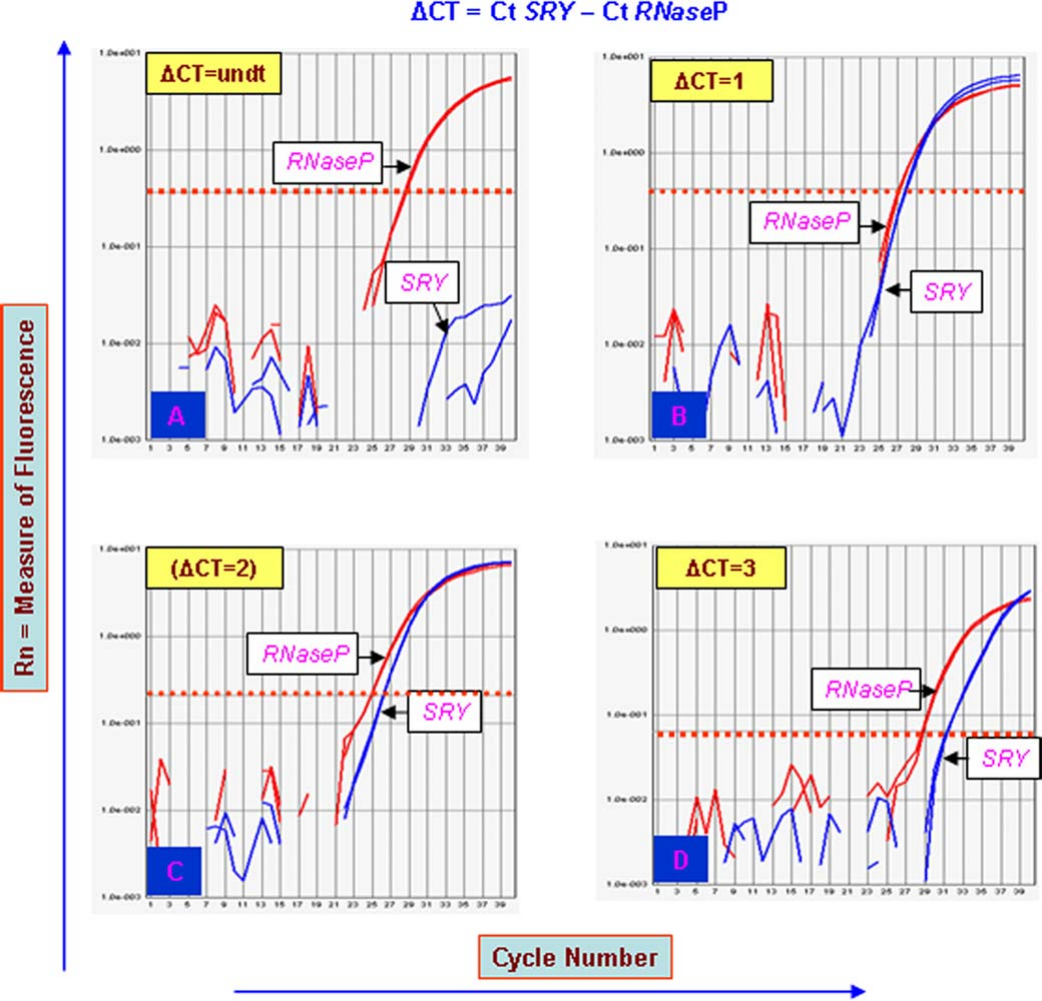
Real Time PCR plots for *SRY* in Turner patients. (A) Due to very low number or absence of cells harboring Y chromosome in Turners' AT2 to AT12, the Ct for *SRY* remained undetermined and thus copy number of the same could not be calculated. (B) Real Time PCR plot of a normal male with ΔCt = 1 corresponding to copies of the *SRY* = 1. (C) and (D) represent plots for additional mosaicisms in the context of percentage of the Y chromosome (and thus for the *SRY* gene) in Turner AT13. The ΔCt values (2 or 4) are unexpected, suggesting that percentage of cells harboring *SRY* is less compared to the ones harboring *RNase*P gene. In Turners AT1 and AT15, ΔCt −3 and −2 respectively, were observed resulting in 16 and 8 copies of the *SRY* gene (not shown).

**Figure 6:**
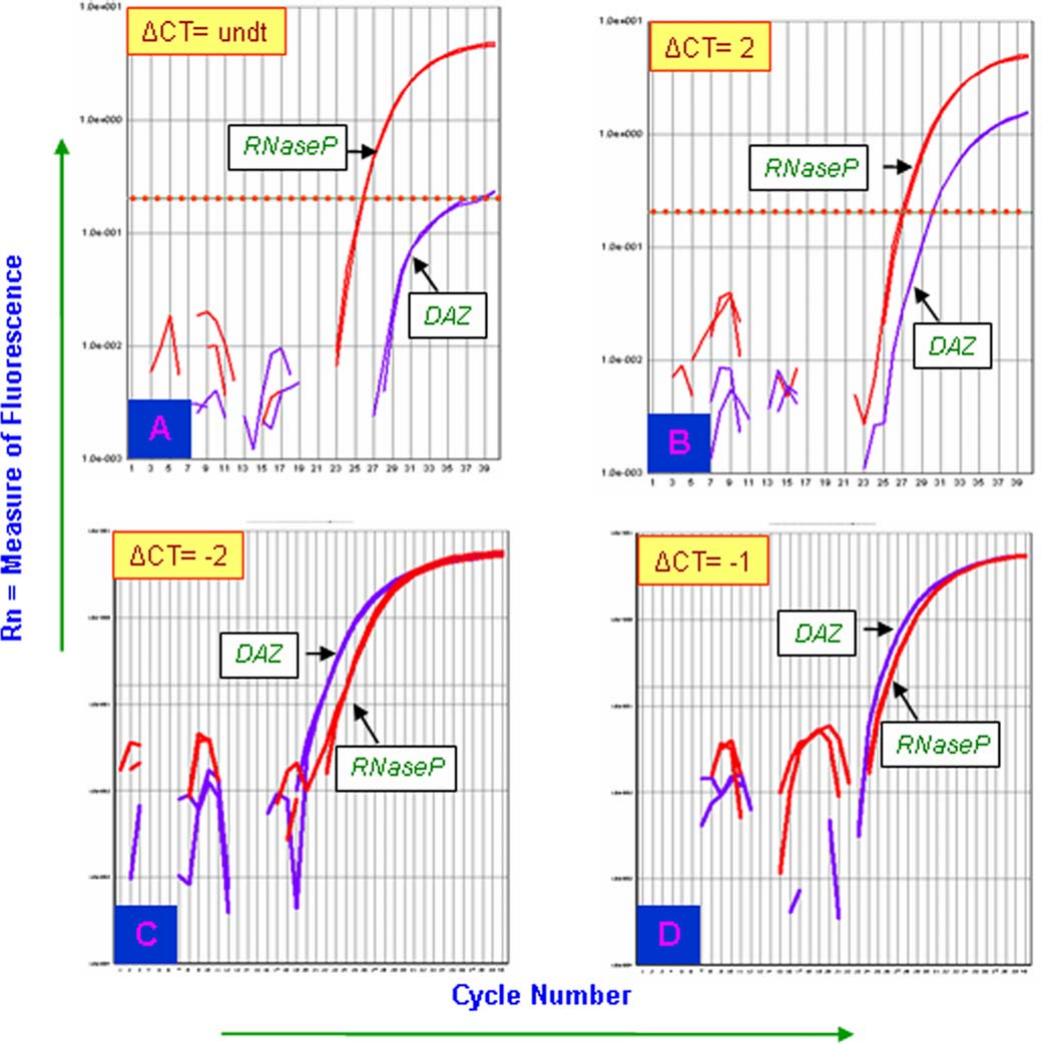
Real Time PCR plots for copy number calculation of the *DAZ* genes in Turner patients. (A) and (B) Similar to the *SRY*, lack of Y chromosome in some Turners resulted a increase in Ct values of *DAZ* genes. (C) Representative plot showing 8 copies instead of 4 of the *DAZ* gene in Turners Patients. (D) Representative plot showing normal 4 copies of the *DAZ* genes with ΔCt = −1.

**Figure 7:**
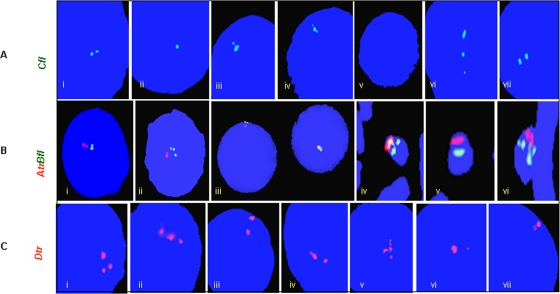
FISH for *DAZ* genes in Turner Patient AT1. The *A*, *B*, *C* and *D* denote *DAZ* probes (supplementary figure 1) and, *tr* and *fl* are for texas red and fluorescein labels, respectively. (A) Note presence of 2 expected signals or a single one owing to overlap in several cells. Some cells lacked signals (v) and others showed 3 signals in place of 2 (vi). (B) Dual probe FISH with *DAZ* probe *A* in red and *B* in green. Expected overlap of the probes *A* and *B* was not observed in most of the cells except a few (iii). Localized *DAZ* signals detected by FISH and multiple copies by Real Time PCR highlight the events of tandem duplication. (C) Analysis of the *AZF*c green amplicons in Turners. Note the presence of all three green amplicons observed in the form of 3 well separated signals. Few cells even showed 2 signals where 1 was of higher intensity compared to that detected in others (vi, vii). This suggests a possible sequence re-modulation or reorganization of the *AZF*c in some percentage of cells in TS patients. The conclusions were based following analyses of 400 metaphase/interphase cells from each Turner Patient.

Turners were assessed for the possible duplication of two candidate genes *DAZ* and *SRY* using TaqMan chemistry and Real Time PCR. In most of the patients, ΔCt (Ct *SRY*/*DAZ* – Ct RNAseP easy) values observed were unexpected. In case of single copy *SRY* and 4 copy *DAZ* genes in a normal male, ΔCt values observed are 1 and −1, respectively [18]. Unexpectedly, ΔCt for *SRY* and *DAZ* gene were >1 in case of TS patients (Figures 5 and 6, Table 2). This is possible only if the copies are less than one, which technically can not be true. The reason behind this was presence of RNAseP gene in all the cells but that of Y chromosome in a small cell population. Even with this mosaicism, few TS patients showed ∼2 to 3 rounds of duplication of the *SRY* and *DAZ* genes (Table 2). FISH conducted for *SRY* and *DAZ* genes showed single localized signal in all the TS patients. This correlated with the events of tandem duplications (See Figures 1 and 7).

**Table 2:** Copy number polymorphism of the *SRY* and *DAZ* genes in the Turner Syndrome patients.

ID	KARYOTYPE	*SRY*		*DAZ*	
		ΔCt	COPIES	ΔCt	COPIES
***p2B***	45,XO 46,XX 46,XY	1–3	Mosaic	−1–0	Mosaic
***p2C***	NA	0–1	1	−1	4
***p4***	45,XO 46,XX 46,XY	5–10	mosaic	0–1	Mosaic
***p65971***	46,XX 46,XY 46,XXXp	−3	16	−2	8
***F- p65971***	NA	0	2	−1	4
***p6697***	NA	^#^Undt	0	Undt	0
***p65972***	NA	−3	16	−0.5–(−1)	Mosaic
***p65975***	NA	−2	8	−1	4
***p17698***	NA	Undt	0	Undt	0
**AT1**	46,XX (10):46,XY (75):45,XO (5):47,XXY (2):47,XYY(3)	−3	16	−1–(−2)	Mosaic
**AT2**	46,XX (77): 46,XY (6):45,XO (14): 47,XXX (3)	Undt	0	Undt	0
**AT3**	46,XX (2):46,XY (3):45,XO (95)	Undt	0	Undt	0
**AT4**	46,XX (0):46,XY (0):45,XO (100)	Undt	0	Undt	0
**AT5**	46,XX (5):46,XY (0):45,XO (95)	Undt	0	Undt	0
**AT6**	46,XX (0):46,XY (0):45,XO (100)	Undt	0	Undt	0
**AT7**	46,XX (5):46,XY (0):45,XO (95)	Undt	0	Undt	0
**AT8**	46,XX (5):46,XY (3):45,XO (92)	Undt	0	Undt	0
**AT9**	46,XX (5):46,XY (5):45,XO (90)	Undt	0	Undt	0
**AT10**	46,XX (5):46,XY (3):45,XO (92)	Undt	0	Undt	0
**AT11**	46, XX (2):46, XY (2):45, XO (96)	Undt	0	Undt	0
**AT12**	46, XX (5):46, XY (3):45, XO (92)	Undt	0	Undt	0
**AT13**	46, XX (40):46, XY (30):45, XO (20)	Undt	0	Undt	0
**AT14**	46,XX (5):46,XY (3):45,XO (92)	Undt	0	Undt	0
**AT15**	46,XX (5):46,XY (80):45,XO (5):47,XXY (2):47,XYY(3)	−2	8	−1–(0)	Mosaic
**NM**	46, XY	1	1	−1	4
**NF**	46XX	Undt	0	Undt	0

Numbers in parenthesis under karyotype denote percentage of the cells with the particular chromosomal constitution. Numbers in parenthesis under karyotype denote percentage of the cells with the particular chromosomal constitution. All the karyotypes were confirmed analyzing ∼400 metaphase chromosome sets per individual. ^#^ Undt” is for undetermined owing to high level of mosaicism.

### Fate of *AZF*c region in Turners

Cosmid probes for DAZ genes and BAC probe used for FISH corresponding to green amplicon demonstrated yet another mosaicism in TS patients. Probe C uncovered expected 2 to a single, widely spaced 3, or no FISH signals. Some cells showed two signals of which one was with higher intensity compared to that of the other suggesting unilocus duplication of the DAZ genes [18]. In the dual color FISH, probe A showed expected 2 or a single signal in most of the cells but probe C again showed multiple signals in certain cell population. Ideally, the signals for probe A and B should overlap owing to their vicinity which was not observed in any of the Turner Patients. This important observation has been explained in a separate report. Significantly, probes C and D uncovered unexpected widely spaced 3 to multiple signals (Figure 7).

### *SRY* mutations

**Figure 8:**
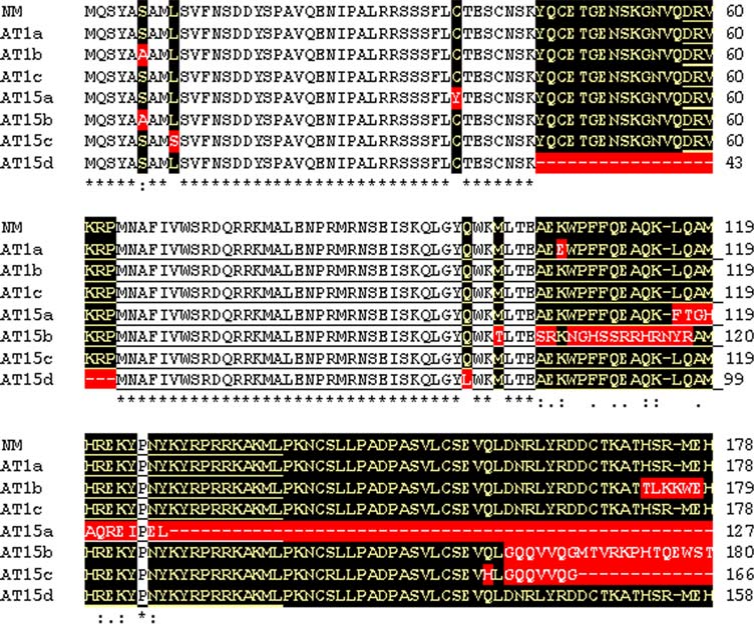
Amino acid changes corresponding to nucleotide sequence of the *SRY* gene in Turners AT1 and AT15. Note the amino acid changes within, upstream and downstream regions of the HMG box which is underlined. Most of these changes detected in the present study were not described earlier.

**Figure 9:**
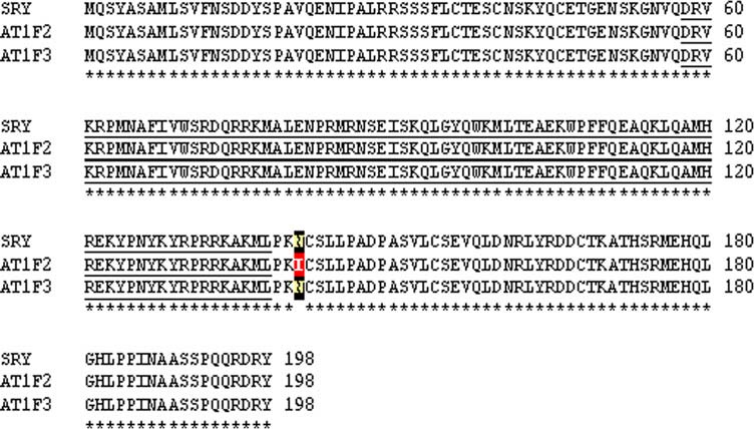
Amino acid changes corresponding to nucleotide sequence of the *SRY* gene in father of a Turner patient AT1. In total ∼ 40 *SRY* recombinant plasmids were sequenced identifying two types of sequences AT1F2 and ATF3. Note that except the change N41I in AT1F2, rest of the amino acid sequence was normal. This highlights *de novo* status of the amino acid changes detected in the Turner AT1.

*SRY* was taken as a candidate gene since the same has been well characterized. This gene was sequenced from all the TS patients who showed positive PCR amplification. All three Turners' positive for *SRY* gene showed normal sequence except a few unclear point nucleotide changes. Mostly, the nucleotide changes were silent except a few affecting the protein sequence. The *in-silico* translation and sequence comparisons showed few well defined amino acid changes upstream, within and down stream of the HMG box. Details of the nucleotide changes of *SRY* gene observed in Turner AT1 are given in figure S2 and corresponding amino acid changes in figure 8. Father of this patient showed a single amino acid change (Figure 9) suggesting that the changes in AT1 were *de novo*, and not inherited from the father.

## Discussion

Turner Syndrome (TS) is globally acknowledged and well defined genetic anomaly, postulated to be an effect of the absence of genes located on the second sex chromosome. However, it is not clear as to how many loci/genes are affected in TS nor do we know whether the abnormal genes/loci are cause or effects. Similarly, there is no information on the type of Y linked genes/loci affected in somatic tissue and gonad of a Turner Patient. Primary focus of the present study was to analyze the fate of the human Y chromosome and its linked loci/genes in TS patients with respect to their possible copy number variation and new type of mutations. This study was undertaken with an ultimate view to uncover consensus changes in the genes/loci to use the resultant information for molecular diagnosis. Extreme mosaicism in terms of the presence or absence of Y chromosome with almost similar hormonal profile and characteristic Turners' features was found to be most astonishing. This suggests that genes involved in control and regulation of hormonal profiles were not affected. Clinically, most accepted karyotype of TS is 45/XO. However, we detected >85% cells harboring intact Y chromosome (46, XY karyotype) in some phenotypically females TS patients. Despite such a large percentage of Y bearing cells, these patients did not represent Swyer syndrome (Gonadal dysgenesis). We hypothesize that in addition to sex chromosomes, there may be several other factors including autosomal genes, responsible for TS phenotype. Other significant part of this study was the absence of dicentrics, marker or ring Y chromosomes in any of the 15 TS patients analyzed though these features were reported to be common in Turner mosaics [16]. Instance(s) of pure XO conceptus cannot be demonstrated unequivocally since chromosome analysis does not uncover the lowest level of Y chromosome mosaicism in lymphocytes or any other tissues. In order to resolve all the ambiguous cases, we used Real Time PCR to monitor copy number status of the genes/loci linked to Y chromosome. This is true also for the other patients suffering from sex chromosome related anomalies such as Turners, Klinefelters, azoospermic and oligospermic ones. It has already been demonstrated that level of mosaicism varies with higher percentage of Y bearing cells in other tissues/organs than that in blood [16]. Gonadal tissue though most important for such analyses is not feasible to be analyzed. Thus, analysis of the blood may fail to allow accessing the actual levels of mosaicism and its correlation with phenotypic sex in TS cases. Copy number analyses demonstrated multiple rounds of tandem duplications wherein two Turners AT1 and AT15 were found to harbor 16 and 8 copies of the *SRY* gene, respectively (Table 2). Based on the karyotype, it is hypothesized that there are varying number of *SRY* genes per cell leading to 16 and 8 copies. Further, two copies of the *SRY* gene detected in father of AT1 suggested that this patient (AT1) did not inherit all the 16 copies instead the same was the result of multiple rounds of tandem duplications. Although no direct evidence is available, these observations suggest that non-disjunction of the Y chromosome and duplications of the linked genes are two independent events. As mentioned earlier, total number of genes/loci affected in case of TS is not known. In addition, involvement of the autosomal genes, their possible up-, down- regulation or genetic imprinting remains allusive in such patients. It would therefore be of relevance if expression level of Y linked genes and possibly autosomal ones are assessed in TS with or without cytogenetically detectable Y chromosome or its mosaicism. Of all the autosomal genes, those that are candidates for testicular functions and hormonal profiles may prove to be attractive targets. Similarly, in-depth mutational analysis of all the candidate genes involved in testicular functions in addition to Y linked loci would also prove to be equally informative. Information on this line is envisaged to be of relevance not only for molecular diagnosis of TS but also for prenatal prognosis and management of clinical cases on routine basis.

### Conclusions

Present study is an attempt to analyze status of the human Y chromosome in patients with Turner Syndrome. Clinical Turner symptoms were common both in females with negligible presence of Y (45, XO) and the ones carrying Y in >85% cells (46, XY). We also infer that in addition to +/− mosaicism of the Y chromosome, Turners may harbor copy number polymorphism of several Y linked genes and possibly that of autosomes. Notwithstanding a great deal of information available in the literature, it is still not proven whether the Y mosaicism observed is a cause or consequence of Turner Syndrome.

## Materials and Methods

### Sample collection and isolation of DNA

Blood samples from all the TS patients were collected from J.N. Medical College, Aligarh Muslim University, Aligarh, India, with the informed consent of patients following strictly the guidelines of the Institute's Ethical and Biosafety Committees. All the patients, except two, were phenotypically females with short stature and webbed neck. TS patients had shield like chest, reproductive sterility and primary amenorrhea (Table 1). DNA isolation was done from the blood samples using standard protocols [19]. Several DNA samples p2b/14, p216c, p4, (p20, p21 Swyer), p65971, p65972, p17698, p6697, p65975, (p75973 and 74, cryptorchidism), p65975 were available from our previous study that were also included [20].

Majority of the Turner patients were illiterate, therefore, their oral consent were obtained. This was facilitated by a recognized local clinician who was also known to them. From the literate people, written consents were obtained following which Institute's Ethical Committee accorded its due clearance. After Institute's Ethical Clearance, no additional approval regarding a particular gene sequence/gene variant was required for publication of the data.

### Estimation of hormonal levels

Level of FSH, TSH, LH and PRL was estimated taking 100 µl serum obtained from each sample by radioimmunoassay using commercial kits from Bhaba Atomic Research Center, Bombay according to supplier's instructions.

### Detection of various Y linked loci by PCR

PCR was performed to amplify *SRY*, *CDY*, *DAZ*, *XKRY*, *DBY*, *UTY*, *CDY2* genes and several STS markers. The amplified product of *SRY* gene from the TS patients was sequenced for its mutational analysis. The *DAZ* genes were assessed for the presence of sequence family variants [18] which was confirmed by sequencing of the PCR products. In addition to direct sequencing, *SRY* fragments were also sequenced in multiples by cloning into pGEMT-easy vector (Promega).

Structural analysis of crucial Y regions was done by selected STSs employing single and multiplex PCRs. The absence of a particular STS was confirmed by repeating the PCR reactions thrice followed by Southern hybridization using amplified PCR product as probe from the normal male. The *AZF*a region was also assessed for possible provirus mediated recombination leading to duplication or deletion [21].

### Copy number assessment of the *SRY* and *DAZ* genes

*SRY* and *DAZ* were chosen as candidate genes for their copy number status. Copy number of was calculated using Real Time PCR and TaqMan/SYBR green assays following procedures described earlier [18], [22].

### Chromosome preparation and fluorescence *in-situ* hybridization (FISH)

Approximately, 400 µl of whole blood was cultured for chromosome preparation following standard protocols [22]. LSI *SRY* (Cat 32-191007) DNA FISH probe for *SRY*/CEP X was purchased from VYSIS (Illinois, USA). FISH probes for DAZ included Cosmids 18E8 for 5′ DAZ, Probe A; 46A6 for 3′DAZ, probe B and 63C9 containing exons 2 through 11, Probe C [23]. For AZFc green amplicon, FISH probe used was BAC RP11-336F2, probe D. The cosmid probes were purchased from Gene service, UK (www.geneservice.co.uk/home) and BAC from Children's Hospital Oakland Research Institute (CHORI). Details of the clones are given in Figure S1. FISH was conducted following standard protocol [18], [22]. Biotynilated anti-fluorescein and anti-texas red antibodies coupled with fluorescein and texas red avidin DCS (Vector Labs) were used in the dual probe FISH experiments. Over 400 interphases/metaphases per individual were analyzed for the presence/absence of the Y chromosome.

## Supporting Information

Figure S1 Details of the FISH probes used for the DAZ genes are listed in the table
(11.64 MB TIF)

Figure S2 Nucleotide sequence polymorphism of the SRY gene in Turners AT1 and AT15.
(14.71 MB TIF)
