# Supplementary figures and images for: Startling Mosaicism of the Y-Chromosome and Tandem Duplication of the SRY and DAZ Genes in Patients with Turner Syndrome
Source: PLoS One. 2008 Nov 24;3(11):e3796. doi: 10.1371/journal.pone.0003796 (PMC2582957; doi:10.1371/journal.pone.0003796)

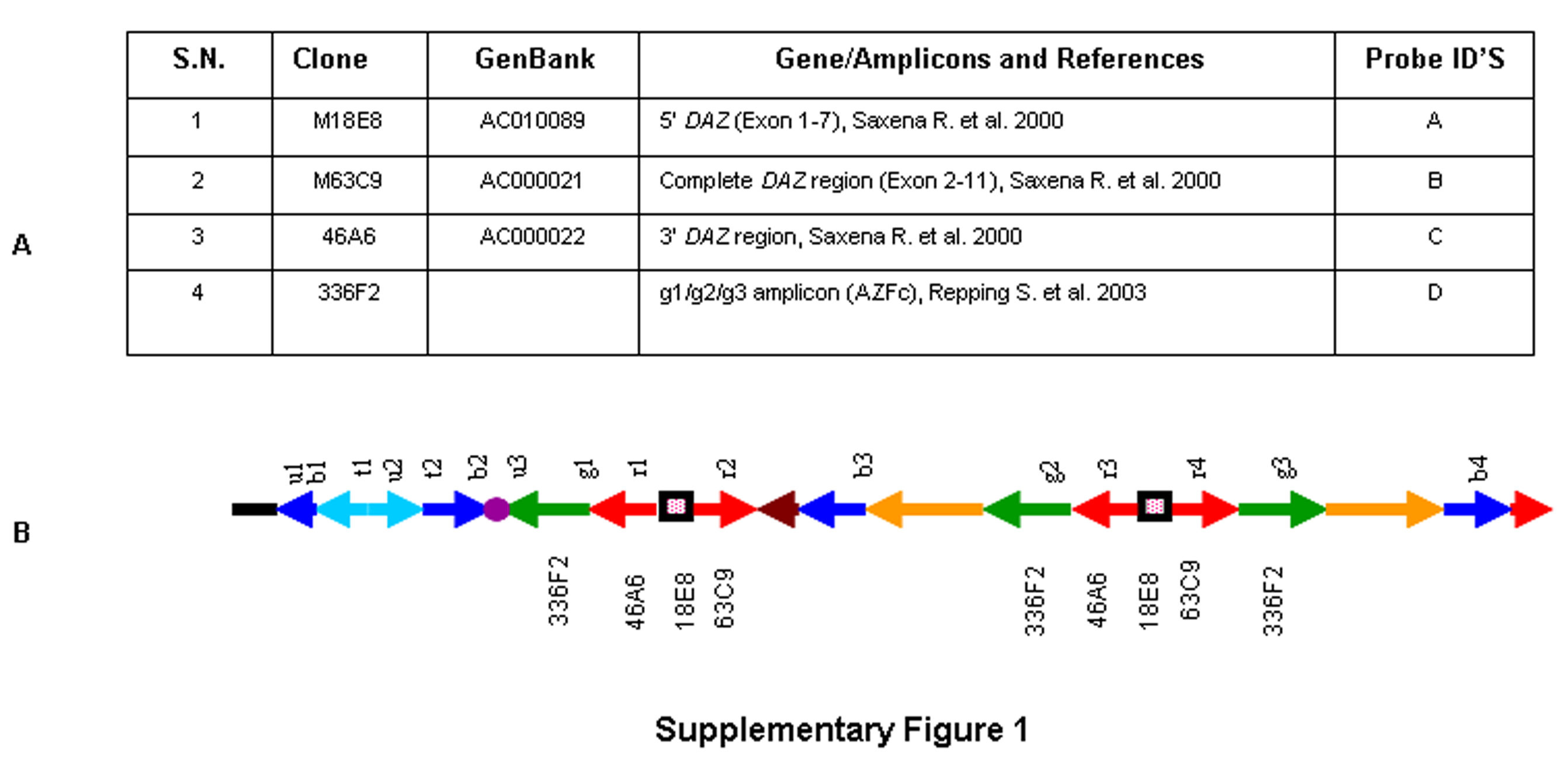

Supplement: Figure S1 — Details of the FISH probes used for the DAZ genes are listed in the table (11.64 MB TIF) [file pone.0003796.s001.tif]

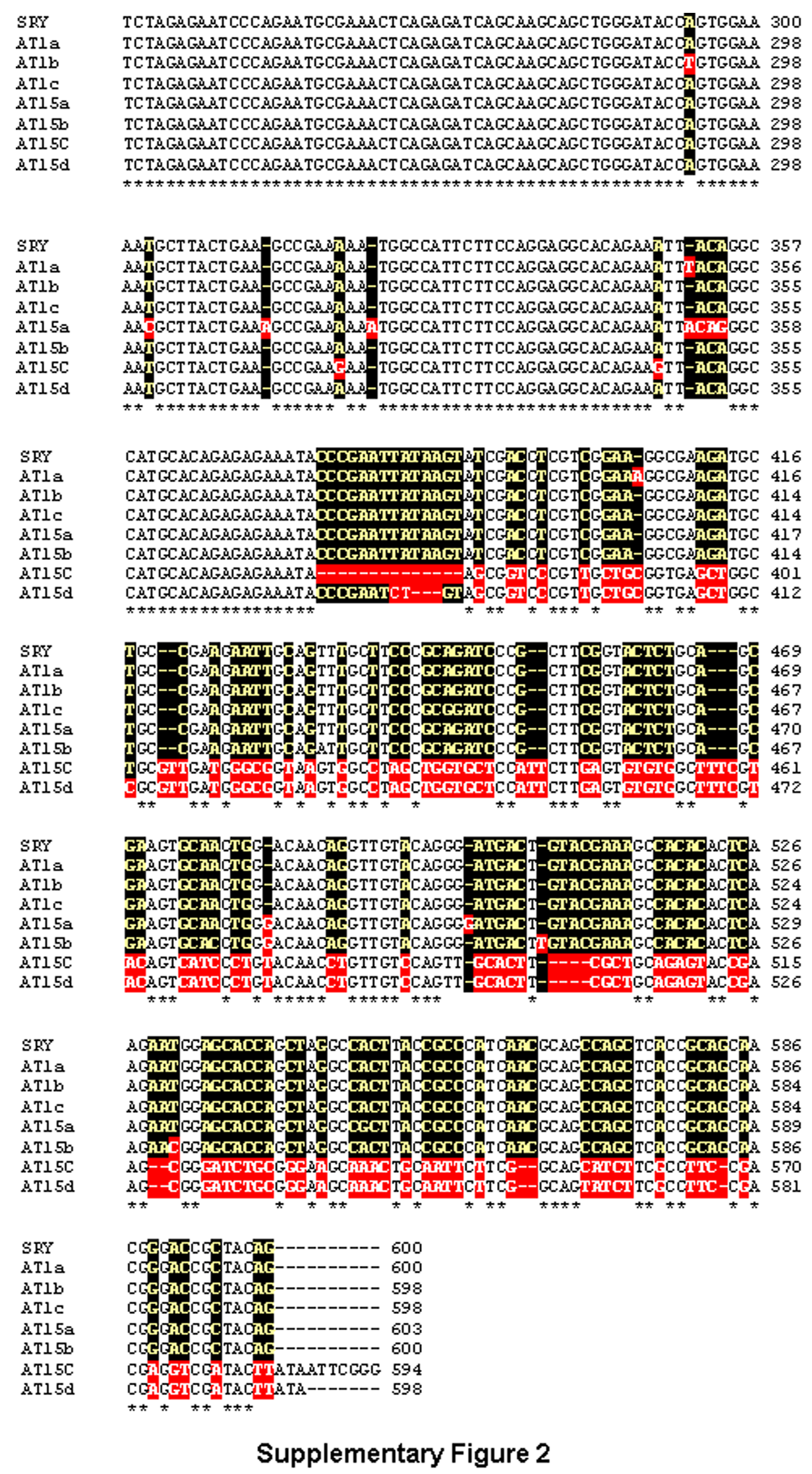

Supplement: Figure S2 — Nucleotide sequence polymorphism of the SRY gene in Turners AT1 and AT15. (14.71 MB TIF) [file pone.0003796.s002.tif]
